# Comparative Study on Online and Offline Teaching for Creative Idea Generation

**DOI:** 10.3389/fpsyg.2022.872099

**Published:** 2022-04-28

**Authors:** Jing Zhang, Ying Dai, Fuxin Zhao

**Affiliations:** ^1^School of Marxism, Hohai University, Nanjing, China; ^2^Faculty of Humanities and Social Sciences, Wenhua College, Wuhan, China

**Keywords:** online teaching, offline teaching, blended teaching, creative idea generation, innovation ability

## Introduction

Innovation is the soul of a nation’s progress and the inexhaustible driving force for a country’s prosperity. Cultivating students’ innovative abilities is an inevitable requirement for China to rejuvenate the country through science and education and build an innovative country, and it is also an important goal of the current education reform (Xi, 2021). In the internet era and the background of global epidemic prevention, the blended teaching model integrating online and offline will become a new normal mode. By comparing the advantages and disadvantages of online teaching and 8 traditional offline teachings, this paper discusses how to make full use of the online and offline blended teaching modes to better promote the cultivation of students’ ability for creative idea generation (Robert and Zeng, 2009).

## Comparison of Online and Offline Teaching for Creative Idea Generation

Each method has its advantages and disadvantages for cultivating students’ creative idea generation (James, 2003; see also Table 1).

**TABLE 1:** Advantages and disadvantages of online and offline teaching modes.

Method	Category	Advantages	Disadvantages
Online teaching	Online teaching	Rich teaching resources, study flexibly and independently, stimulate learning interest, enhance learning initiative, develop globalize vision, cultivate creative idea generation and ability	Backward teaching ideas of some teachers, inexplicit consciousness of initiative and innovation of some students, lack of practical training conditions
Lecture		Stimulate learning interest and motivation, understand and internalize knowledge, generate new knowledge, create new ideas	Cramming method of teaching, limited level of students’ speech and thinking brings passive learning, influences learning effect and creativity
5WH	Offline teaching	Open minds, clarify ideas, guide grasping the essence of the problem, deep explore, produce a new idea	Fixed analysis mode, difficult to analyze complex problems, not conducive to divergent thinking
Case		Vivid, specific, intuitive, easy to understand; train creative idea generation by analyzing various cases	Difficult to find suitable cases for teaching, unsuitable cases limit innovation consciousness
Discussion		Offer a chance to speak and discuss, give full play to the initiative, active thinking, promote inspiration, generate new ideas	The lax organization makes some students forget the new idea has been created, brings out evaluation anxiety and cognitive interference, etc.
Mind mapping		Establish a remote association and the internal connection between each knowledge point, form a systematic knowledge system, improve understanding and memory ability, forms new ideas	Teachers and students are required to have a high level of cognition, or it’s a mere formality, limiting the expansion and innovation of the creative idea
Reverse thinking		Change the way of thinking, improve flexibility and creativity	Shallow thinking and no creative idea if failed to grasp the deep problems or essence of things
Role-playing		Arouse curiosity and interest, from passive learning to active inquiry, enhance interactive teaching, improve creative idea generation, shape creative personality	Participants’ number is limited, innovation motivation will be affected if no chance to attend or with low participation
Practice		Consolidate and deepen theoretical knowledge, link knowledge, and practical problems through practice, solve problems in practicing, generate new ideas	Creative idea generation will be affected because of time, place, organization, funding, security, etc.

Online teaching transfers the teaching content of traditional classrooms to the network by using online teaching platforms, network chat tools, video conferences, and other forms. The teaching model includes network broadcast teaching, video network teaching, large-scale online open class, and other forms. It can provide rich learning resources (Day and McCulloch, 1996), flexible and independent ways for teachers to teach and students to learn, which helps stimulate students’ learning interest and initiative, and develop an international vision. All of these contribute to cultivating students’ creative idea generation (Aggarwal et al., 2021). However, these factors may affect the cultivation of students’ creative idea generation, such as some teachers’ backward teaching ideas (Oksana Andriivna et al., 2021), some students’ inexplicit consciousness of initiative and innovation, and the lack of practical training conditions.

Traditional offline teaching has been used so far and formed multiple teaching modes such as lecture method, 5WH (what, where, when, who, why, how), case method, discussion method, mind-mapping method, reverse thinking, role-playing method, practice method, etc. It offers the chance for students to receive face-to-face guidance from teachers, which contributes to enhancing the emotional communication between them, cultivates students’ creative idea generation, and shapes their creative personality, while teachers pass on knowledge to students (Cao, 2015). However, offline teaching is limited by time and space in teaching and communication between teachers and students. It may also lead to students being not updated on the latest academic trends at home and abroad because the information that teacher passed to students is limited, the learning resources are not updated in time, which make it difficult to stimulate students’ endogenous motivation and learning interest, and is not conducive to the cultivation of students’ creative idea generation.

The following will analyze the advantages and disadvantages of cultivating students’ creative idea generation, taking examples as case method, mind mapping, role-play, and practical method.

The case method is based on certain teaching objectives and specific teaching cases. It is vivid, specific, intuitive, and easy to understand. Focusing on a practical problem that needs to be solved, students can be “immersive” through various cases to find ideas and solutions to deal with the problems, to train students’ creative idea generation and creativity to deal with various emergent problems (Gu et al., 2020). However, it’s difficult to find suitable cases for teaching points. At the same time, students are guided to find the point of connection between the problem and teaching and put forward feasible measures to solve the problem creatively.

Mind-mapping uses the skills of paying equal attention to pictures and texts, the relationship between all levels of the theme is represented by the hierarchical map of mutual subordination and correlation, and the theme’s keywords and images, colors, and other memory links are established to make the thinking process visible. It can quickly help students establish a remote association and the internal connection between each knowledge point, build a knowledge network, form a systematic knowledge system, give full play to the left and right brain functions, improve students’ understanding and memory ability, and help students’ creative idea generation. It requires both teachers and students to have a high level of cognitive ability, otherwise, it will become a mere formality (Huang et al., 2020), or limit the expansion and innovation of creative ideas.

Role-play allows students to think and experience real emotions in a specific role in a specific situation. Role transformation can arouse students’ curiosity and interest, make students change from passive learning to active inquiry, enhance interactive teaching, improve students’ creative idea generation, and it is also conducive to the shaping of creative personality during the role-play (Azmi et al., 2018). However, it is limited by the number of participants, and for the student who has no chance to attend or with low participation, their innovation motivation will be affected.

The practical method constructs a student-centered teaching activity with education, creativity, and practicality, and it encourages students to actively participate, and think deeply, to promote the students’ overall quality and all-around development (Shi, 2013). It helps students consolidate and deepen their theoretical knowledge, offer the chance to give full play to their subjective initiative, link knowledge, and practical problems through practice, realize a qualitative leap from “understanding” to “solving problems,” and improve their cognition, innovation, practice, and other comprehensive ability and quality. However, time, place, organization, funds, safety, and other issues should be taken into consideration, or it will affect students’ creative idea generation and creativity.

## Blending Teaching Mode

During the Coronavirus disease 2019 (COVID-19) outbreak in 2020, 94% of the 1.6 billion students around the world studied online. Until January 2021, there are still 700 million students worldwide who are still learning online (The World Bank, 2021). In China, hundreds of thousands of schools, 280 million students, and 17 million teachers conducted online teaching (China Education Network, 2020).

**FIGURE 1:**
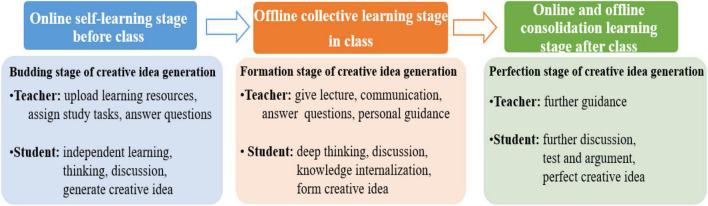
Creative idea generation diagram of blended teaching.

As global epidemic prevention and control have become normalized, blended teaching has become the most commonly used teaching mode in schools. It is not a simple combination of online and offline teaching modes, but an organic integration of multiple teaching modes and different teaching objects, teaching contents, and teaching needs (Van Doorn and Van Doorn, 2014; Bao et al., 2021). The students’ creative idea generation is mainly reflected in three stages in blended teaching (Maggio et al., 2012; see also Figure 1).

The first stage is the online self-learning stage before class, it is also the budding stage for creative idea generation when students study independently under the teachers’ guidance. Teachers prepare and upload learning resources to the online platform and set some learning tasks according to the teaching goal. Students learn autonomously, communicate and discuss with teachers puzzling questions or thoughts online, and generate new ideas (Bai et al., 2020).

The second stage is the offline collective learning stage in class, it is also the formation stage of creative idea generation under the interaction between teachers and students. Teachers explain students’ common problems by teaching method, discussing method, case method, role-play, etc., and instruct students’ different problems individually. Students deepen the understanding of learning content, complete the internalization of knowledge, form their creative idea generation according to teachers’ teaching and guidance, and through exchanging and discussing with classmates. This is also the process that students learn from the traditional individual learning toward group learning, creating knowledge and collective wisdom (Aguilar-Zambrano and Trujillo, 2017).

The third stage is the online and offline consolidated learning stage after class, it is also the perfection stage of creative idea generation by the effort of teachers and students together. Students will process and improve the creative ideas through further discussion with teachers and other students, and test and verify the correctness and scientificity of the generated idea.

A recent study by Bao et al. (2021) on the 62,650 samples collected from a *Survey on Teaching Quality and Student Development in National Colleges and Universities* conducted by the School of Education of Peking University in 2017 and 3,537 samples from *a Survey on Online Course Learning of Undergraduate Students* in 2020, we can see that blended teaching has the advantages of online and offline teaching, and it complements the shortcomings of single online and offline teaching effectively. It does not only break the limitation of time and space of offline teaching, but it also makes up for the separation of individual education and the lack of emotional interaction caused by online teaching. It provides favorable conditions for cultivating students’ critical thinking and creative ability and greatly improves students’ creative idea generation.

Firstly, blended teaching embodies the concept of teacher-centered to student-centered and teacher-led (Duan et al., 2022). Teachers pay attention to students’ learning situations online in real-time and answer their questions in time, and give further guidance offline, which is conducive to enhancing the affection between teachers and students. communication and interaction can be online at any time between teachers and students, or classmates, which reduces students’ psychological pressure such as constriction, tension, and other scruples in the classroom. These help to improve students’ learning initiative and enthusiasm (Wong, 2019), tap their inner potential, make their thinking more active, and help students to generate creative ideas by changing from knowledge-oriented to ability-oriented.

Secondly, teachers can choose the different teaching content, teaching method, and teaching way according to students’ characteristics, and make personalized teaching plans for students. For example, in foreign language teaching, teachers can choose simple and interesting content and adopt lecture methods to stimulate their learning interest for students with little interest in learning and weak foundation; while teachers can set corresponding difficult teaching content and use diversified teaching methods, to better cultivate their innovative consciousness and creative ability to the students who like foreign languages and have a good foundation.

Thirdly, teachers adopted diversified teaching methods according to different courses. For example, for some courses that must be completed through experiments or practices, teachers can carry out theoretical teaching online and experimental operations offline, to arrange teaching time reasonably and effectively, so that students can complete creative idea generation during the process of online and offline teaching.

## Conclusion

Overall, both online teaching and offline teachings have advantages and disadvantages. In the internet era and the background of global epidemic prevention, blended teaching, which combines multi-teaching, is an inevitable choice, and it is more effective than single teaching for students’ creative idea generation. At present, many educators are actively practicing and exploring how to maximize the advantages of the mixed-use of various teaching modes, improve students’ subjective initiative, enhance their innovative spirit and creative idea generation ability, and cultivate them into excellent talents with the high-quality innovative ability and comprehensive quality.

## Author Contributions

All authors listed have made a substantial, direct, and intellectual contribution to the work, and approved it for publication.

## Conflict of Interest

The authors declare that the research was conducted in the absence of any commercial or financial relationships that could be construed as a potential conflict of interest.

## Publisher’s Note

All claims expressed in this article are solely those of the authors and do not necessarily represent those of their affiliated organizations, or those of the publisher, the editors and the reviewers. Any product that may be evaluated in this article, or claim that may be made by its manufacturer, is not guaranteed or endorsed by the publisher.

## References

[B1] Aggarwal V. Hwang E. H. Tan Y. (2021). Learning to be creative: a mutually exciting spatiotemporal point process model for idea generation in open innovation. *Inf. Syst. Res.* 32 1214–1235. 10.1287/isre.2021.1020 19642375

[B2] Aguilar-Zambrano J. J. Trujillo M. J. (2017). “Factors influencing interaction of creative teams in generation of ideas of new products: an approach from collaborative scripts,” in *Proceedings of the 2017 Portland International Conference on Management of Engineering and Technology (PICMET 2017)*, Portland, OR, 1–11. 10.23919/PICMET.2017.8125391

[B3] Azmi N. H. Surat S. Marzuki M. A. Yusoff A. N. Rahman S. (2018). Effects of idea generation module on students’ creative self-efficacy. *Adv. Sci. Lett.* 24 8463–8466. 10.1166/asl.2018.12589

[B4] Bai X. Wang X. Wang J. X. Tian J. Ding Q. (2020). “College students’ autonomous learning behavior in blended learning: learning motivation, self-efficacy, and learning anxiety,” in *Proceedings of the 2020 International Symposium on Educational Technology (ISET 2020)*, Bangkok, 155–158. 10.1109/ISET49818.2020.00042

[B5] Bao W. Chen D. C. Wang J. (2021). Research on online and traditional learning paradigm and teaching effectiveness in the post COVID-19 era. *China Educ. Technol.* 6 7–14.

[B6] Cao P. J. (2015). *Rebooting Creativity: The Rules of Innovation for the Internet Age.* Beijing: Beijing Jiaotong University Press.

[B7] China Education Network (2020). *Review of Education Informatization under the Epidemic of 2020.* Available online at: https://www.edu.cn/info/zt/fk/202102/t20210204_2075196.shtml (accessed February 3, 2022).

[B8] Day J. G. McCulloch I. D. (1996). Algae on the internet. *J. Appl. Phycol.* 8 205–210. 10.1007/BF02184973

[B9] Duan J. J. Fan Y. N. Zhao Q. H. (2022). Practices of student-generated content (SGC) pedagogy from different teaching presence perspectives: based on interactive networks and social knowledge constructs. *J. Distance Educ.* 1 61–71. 10.15881/j.cnki.cn33-1304/g4.2022.01.006

[B10] Gu C. H. Han M. Li C. Bie Z. Tan Y. Y. Xue Y. K. (2020). The effect of environmental cues and motivation on creative idea generation. *Creat. Innov. Manag.* 29 581–596. 10.1111/caim.12403

[B11] Huang L. P. Liu B. Ying Y. T. Li S. S. (2020). The application of problem-oriented mind mapping in the teaching of pharmacology under the environment of MOOCs. *J. Yichun Univ.* 12 101–105.

[B12] James W. Y. (2003). *A Technique for Producing Ideas.* New York, NY: McGraw-Hill Press.

[B13] Maggio L. A. Davies K. J. Allee N. Beattie J. Berryman D. Littleton D. (2012). Literature searching in medical education: online tutorial development from idea to creation. *Med. Ref. Serv. Q.* 31 372–382. 10.1080/02763869.2012.724274 23092415

[B14] Oksana Andriivna B. Olena Vasylivna K. Lopushanskyy V. Valeriia Mykhaylivna S. Yukhymets S. (2021). Psychological difficulties during the covid lockdown: video in blended digital teaching language, literature, and culture. *Arab World Engl. J.* 19 172–182. 10.24093/awej/covid.13

[B15] Robert J. S. Zeng P. P. (2009). *The Psychology of Creativity: Awakening our Innate Creative Potential.* Beijing: China Renmin University Press.

[B16] Shi W. (2013). On the university practical teaching system. *J. High. Educ.* 7 61–64.

[B17] The World Bank (2021). *Urgent, Effective Action Required to Quell the Impact of COVID-19 on Education Worldwide.* Available online at: https://www.shihang.org/zh/news/immersive-story/2021/01/22/urgent-effective-action-required-to-quell-the-impact-of-covid-19-on-education-worldwide (accessed February 5, 2022).

[B18] Van Doorn J. R. Van Doorn J. D. (2014). The quest for knowledge transfer efficacy: blended teaching, online and in-class, with consideration of learning typologies for non-traditional and traditional students. *Front. Psychol.* 5:324. 10.3389/fpsyg.2014.00324 24860517 PMC4029015

[B19] Wong R. (2019). Basis psychological needs of students in blended learning. *Interact. Learn. Environ.* 12, 1–15. 10.1080/10494820.2019.1703010

[B20] Xi J. P. (2021). Fully Implement the Strategy of Strengthening the Country Through Talent for a New Era and Accelerate Efforts to Build China into a Major Talent Center and Innovation Hub in the World. Available online at: http://www.qstheory.cn/dukan/qs/2021-12/15/c_1128161060.htm (accessed January 28, 2022).

